# The morphology, biomechanics, and physiological function of the suboccipital myodural connections

**DOI:** 10.1038/s41598-021-86934-4

**Published:** 2021-04-13

**Authors:** Yue Ma, Wei Tang, De-Zheng Gong, Xing-Yi Li, Jing-Hui Zhang, Jia-Hui Sun, Bing Wang, Ying Zhang, Yu-Xiao Chen, Zhi-Hong Zhang, Nan Zheng, Chukwuemeka Samuel Okoye, Yan-Yan Chi, Cheng-Wei Wu, Sheng-Bo Yu, Hong-Jin Sui

**Affiliations:** 1 The Second Affiliated Hospital of Dalian Medical University, Dalian, 116027 Liaoning People’s Republic of China; 2 Department of Anatomy, College of Basic Medical Science, Dalian Medical University, 9 West Section, Lushun South Road, Dalian, 116044 People’s Republic of China; 3 Medical Foundation Experiment Teaching Center, College of Basic Medical Science, Dalian, Liaoning 116011 People’s Republic of China; 4 The First Affiliated Hospital of Dalian Medical University, Dalian, 116011 Liaoning People’s Republic of China; 5 Department of Anesthesiology, Dalian Medical University, Dalian, 116044 Liaoning People’s Republic of China; 6 Department of Engineering Mechanics, State Key Lab of Structural Analysis for Industrial Equipment, Faculty of Vehicle Engineering and Mechanics, Dalian University of Technology, Dalian, 116024 Liaoning People’s Republic of China

**Keywords:** Physiology, Anatomy, Medical research, Neurology

## Abstract

The myodural bridge (MDB) connects the suboccipital musculature to the spinal dura mater (SDM) as it passed through the posterior atlanto-occipital and the atlanto-axial interspaces. Although the actual function of the MDB is not understood at this time, it has recently been proposed that head movement may assist in powering the movement of cerebrospinal fluid (CSF) via muscular tension transmitted to the SDM via the MDB. But there is little information about it. The present study utilized dogs as the experimental model to explore the MDB’s effects on the CSF pressure (CSFP) during stimulated contractions of the suboccipital muscles as well as during manipulated movements of the atlanto-occiptal and atlanto-axial joints. The morphology of MDB was investigated by gross anatomic dissection and by histological observation utilizing both light microscopy and scanning electron microscopy. Additionally biomechanical tensile strength tests were conducted. Functionally, the CSFP was analyzed during passive head movements and electrical stimulation of the suboccipital muscles, respectively. The MDB was observed passing through both the dorsal atlanto-occipital and the atlanto-axial interspaces of the canine and consisted of collagenous fibers. The tensile strength of the collagenous fibers passing through the dorsal atlanto-occipital and atlanto-axial interspaces were 0.16 ± 0.04 MPa and 0.82 ± 0.57 MPa, respectively. Passive head movement, including lateral flexion, rotation, as well as flexion–extension, all significantly increased CSFP. Furthermore, the CSFP was significantly raised from 12.41 ± 4.58 to 13.45 ± 5.16 mmHg when the obliques capitis inferior (OCI) muscles of the examined specimens were electrically stimulated. This stimulatory effect was completely eliminated by severing the myodural bridge attachments to the OCI muscle. Head movements appeared to be an important factor affecting CSF pressure, with the MDB of the suboccipital muscles playing a key role this process. The present study provides direct evidence to support the hypothesis that the MDB may be a previously unappreciated significant power source (pump) for CSF circulation.

## Introduction

The MDB is a complex anatomical structure that physically connects the cervical spinal dura mater (SDM) to the suboccipital muscles, including the rectus capitis posterior minor (RCPmi), the rectus capitis posterior major (RCPma), and the obliquus capitis inferior (OCI) while passing through the posterior atlanto-axial and atlanto-occipital interspaces^1–5^. Recently, several published studies have identified the existence of the MDB in mammals (porpoises, macaques, dogs, cats, mice, and rabbits)^6^, birds^7,8^ and reptiles^9^, that the MDB is an evolutionary conserved physical structure that is involved in important physiological functions.

Concerning the functionality of the MDB, it has recently been proposed that contraction of the suboccipital muscles, produced during head movement, might assist in resisting SDM in-folding^1^. Moreover, that the suboccipital muscles, along with MDB might play an essential role in transmitting cervical proprioception by monitoring tensional changes in the cervical SDM and effecting a rapid adjustment of the head position^10^. Furthermore, as the MDB might prevent the cervical SDM from folding inward the spinal cord during head and neck movements thus preventing the impedance of cerebrospinal fluid (CSF) flow in the subarachnoid space and cisterna magna^2,4,11^. Other recent studies have postulated that during head movement, the MDB would pull on the cervical SDM producing changes in the volume and pressure of the CSF located in the cisterna magna and subarachnoid space of the upper cervical spine, consequently promoting the circulation of the cerebrospinal fluid at the occipito-cervical junction^12–14^. A recent study using the PC cine of MRI evidenced a significant increase in the net flow of cerebrospinal fluid in the cephalic direction after head rotation^15^, providing indirect evidence for the hypothesis that the MDB serves as one of the power sources for CSF circulation.

Clinically, dysfunctional suboccipital musculature was observed to be related to various types of headaches^16–20^. However, its mechanism is still unknown up to now. The basic research on the function of the myodural bridge is very important for the study of the mechanism of clinical disease.

To test the proposed “cerebrospinal fluid circulation dynamic hypothesis” relating to the myodural bridge, the present study explored the effects of suboccipital muscles contractions and head movements on the CSF pressure and the potential role of MDB.

## Materials and methods

### Experimental subjects

Seventy-three healthy adult Beagle dogs (Both male and female, body weight 10–12 kg, body length 650–770 mm) were provided after experimental teaching of surgery for medical students by the Experimental Animal Center of Dalian Medical University. All animal experiments conformed to ethical requirements of the school.

### Investigations of the morphology of MDB

#### Gross anatomic dissection in the nuchal regions

Ten Beagle dogs were euthanized. Afterwards, the nuchal region of each dog was dissected, layer by layer, to explore the subocciptal musculature.

##### Dissection at the level of the dorsal atlanto-occipital interspace

The rectus capitis dorsal minor (RCDmi) muscle was fully exposed. Fibrous connections between the RCDmi and the dorsal atlanto-occipital membrane (DAOM) were observed as well as fibrous connections between the DAOM and the upper cervical SDM.

##### Dissection at the level of the dorsal atlanto-axial interspace

The short head of the rectus capitis dorsal major (RCDma) and the obliquus capitis inferior (OCI) muscles were exposed integrally. Fibrous connections between these two muscles and the dorsal atlanto-axial membrane (DAAM) were observed as well fibrous connections between the DAAM and the upper cervical SDM.

#### Histological study of connections between the dorsal occipital muscles and the SDM

Utilizing six of the euthanized Beagle dogs, tissue-blocks from the sub occipital region were prepared, which included the suboccipital muscles, the cervical dura mater, and tissues between them at the level of the dorsal atlanto-occipital interspace or dorsal atlanto-axial interspace. These tissue blocks were conventionally sectioned and stained with Masson trichrome and HE. The fibrous connections among the suboccipital muscles, DAOM (or DAAM) and SDM were observed and photographed under the light microscope.

#### Ultrastructural investigation of connections between the dorsal occipital muscles and SDM under the scanning electron microscope (SEM)

Tissue blocks taken from eight of the euthanized Beagle dogs at the level of the dorsal atlanto-occipital interspace, consisted of the RCDmi, the DAOM and the SDM were dissected out.

At the level of the dorsal atlanto-axial interspace, tissue blocks consisting of the OCI, the short head of the RCDma, the DAAM, and the SDM were obtained.

These specimens all underwent conventional preparation for SEM evaluations. The fibrous connections between the DAOM (or DAOM) and the SDM at the dorsal atlanto-occipital and atlanto-axial interspaces were observed and photographed under the SEM.

### Biomechanical investigation of connections between the dorsal occipital muscles and the SDM

#### Specimen preparation

**Figure 1:**
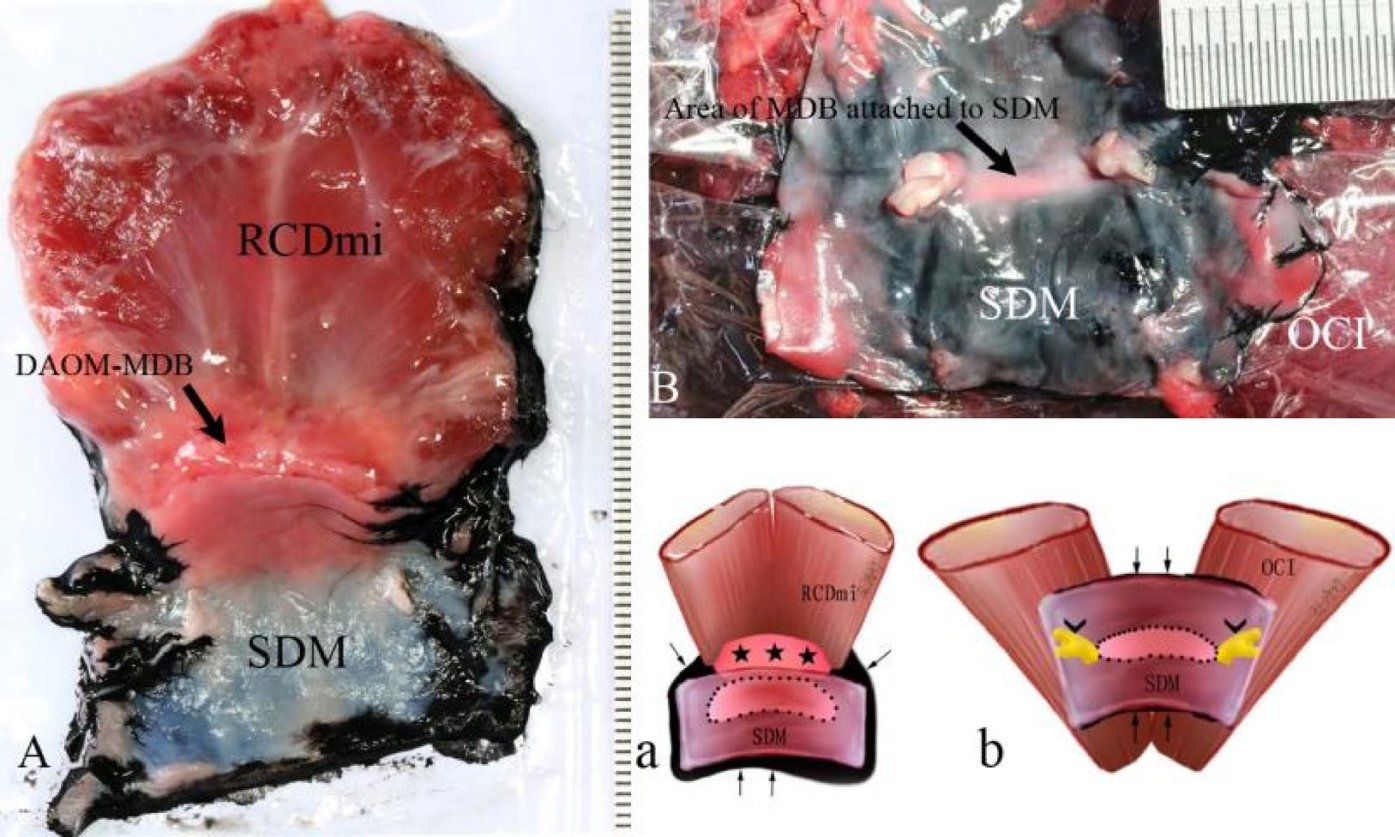
Diagram of the method for showing the connected area between the MDB and the SDM. The contour of the area of the MDB attached to the SDM was showed by infiltrated black ink. Figure 1A: The complex of RCDmi-MDB-SDM located at the dorsal atlanto-occipital interspace; Fig. 1B: The complex of OCI-MDB-SDM located at the dorsal atlanto-axial interspace; Fig. 1a: Illustration for Fig A; Fig. 1b: Illustration for Fig. 1B. Thin black rrows: black ink infiltrated into the narrow gap between the SDM and muscles. Dot line: the connected area between the MDB and the SDM showed up by black ink infiltrating. Stars: DAOM and MDB. Arrowheads: Roots of the spinal nerve.

Tissue blocks were taken from the euthanized the Beagle dogs (N = 13) were utilized for this experiment. In the dorsal atlanto-occipital interspace, the specimens were intact tissue blocks containing the RCDmi, the DAOM, the SDM, and the fibrous connections (Fig. 1A,a). The dorsal atlanto-axial interspace, test samples were prepared containing the short head of the RCDma, the OCI, the DAAM, the SDM, and fibrous connections among them (Fig. 1B,b).

#### Measurement of the connected areas between the MDB and SDM

The SDM, the DAOM (or DAAM), and the muscles (RCDmi and OCI) were placed on a plastic film while maintaining their original *in-vivo* relationships. Ink (Hero 234 Advanced Carbon Ink, Shanghai Ink Factory, China) was then dropped into the narrow gap between the SDM and the other tissues to evidence the contour of the connected area between the MDB and the SDM (Fig. 1). After staining the specimens the specimens were photographed and the connected areas between the MDB and the SDM were measured using Adobe Photoshop (URL:http://m.xitongtiandi.net/soft_tx/5244.html, version number:13.0 (13.0 20120315.r.428 2012/03/15:21:00:00) × 64) software.

#### Tensile testing

**Figure 2:**
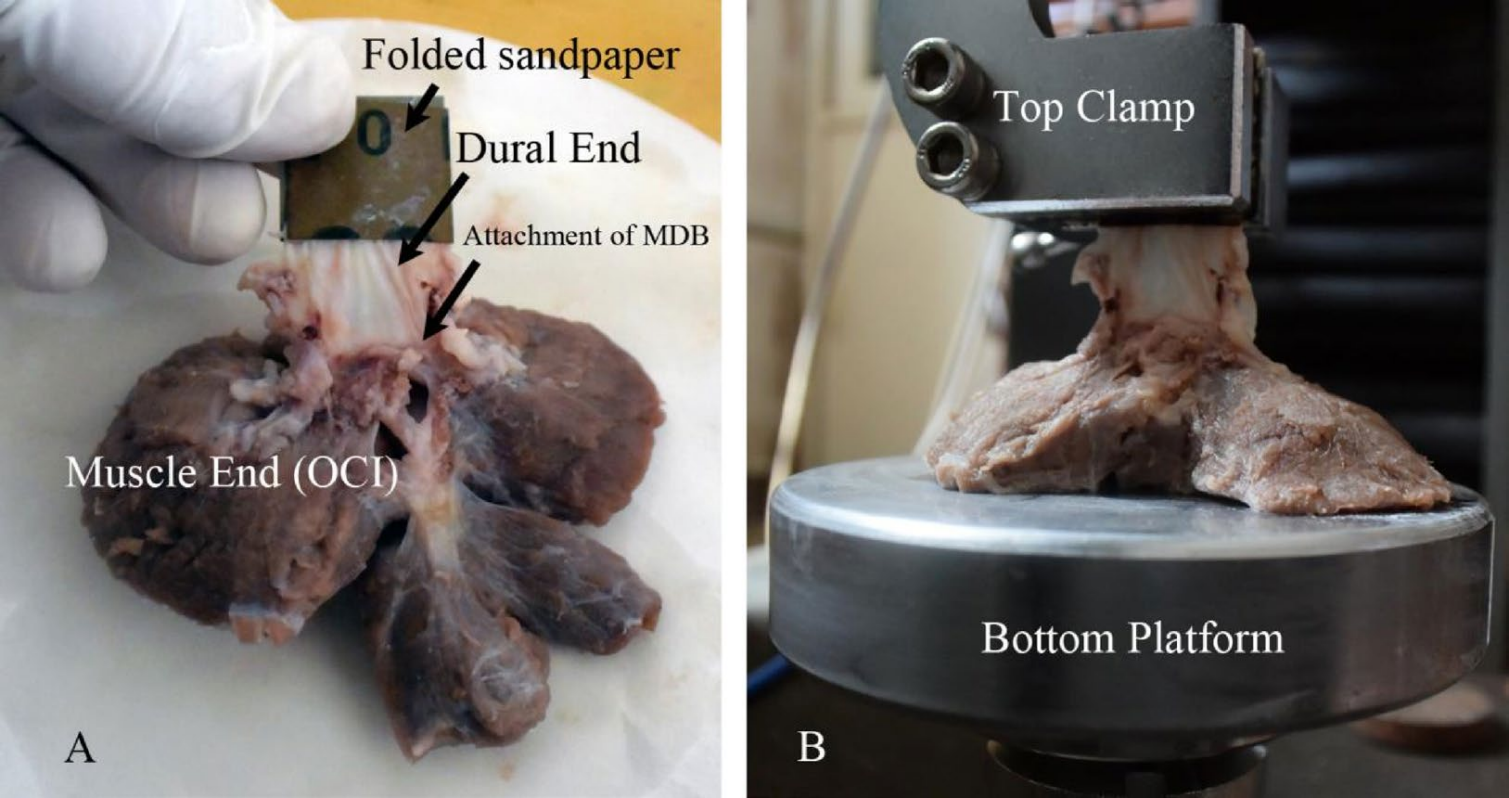
Illustration of preparation (**A**) and fixation (**B**) of the test specimen.

The fresh specimens were sealed in airtight bags and stored at 4 degree (C). Before measurement the specimens were left at room temperature for half an hour. Uniaxial tension experiments were performed at room temperature within the post mortem time of 10hrs. The specimen was fixed on the test rig as shown in Fig. 2. The surface of muscle end of the specimen was glued (Cyanoacrylate Super Glue, Deli Group Co., Ltd., China) onto a bottom platform, and the dural end of the specimen was glued in a folded sandpaper and clamped into the top clamp, which mounted on to a 50 N capacity load cell with sensitivity of 1mN (BAB S type aluminum weighing sensor, BAB-XS-5 M, Transcell Technology Inc., USA), which was attached to the actuator of a 20 kN CMT-4204 electronic universal testing machine (New Sansi Material Testing Co., Ltd, China). No preconditioning was conducted and only one loading cycle was executed on each specimen. The sampling frequency for both the force and displacement data was 100 Hz. Following this, the force was set at zero. The top clamp was raised slowly so as to just stretch the dura mater tight. Then the displacement was set at zero. After this, the top clamp moved with a pre-set velocity (10 mm/min) and the force–displacement curve until failure was recorded. The ultimate tensile strength of the MDB was calculated utilizing the ratio of the fracture force with the area of the connection between the MDB and the SDM.

### Cerebrospinal fluid pressure measurement during head movements or electrical stimulation of the suboccipital muscles

Pre-operation preparation: 33 animals were used for this aspect of the study. 3% Nembutal was injected into the animal intraperitoneally for anesthesia with tracheal intubation.

Cerebrospinal fluid pressure monitoring: The puncture needle was inserted into the subarachnoid space through the dorsal space between L4 and L5. When cerebrospinal fluid (CSF) appeared at the opening of the puncture needle, a catheter was inserted 24 cm cranially into the spinal canal and then connected to a blood pressure sensor (PT-102, Sensitivity:100 μV/mmHg, Resolution: 200 mmHg/(65536/2) = 0.006 mmHg) to record the CSF pressure by BL-420F biological data acquisition and analysis system (ChengDu Techman SoftWare CO., LTD. China) during the experiment.

Blood pressure monitoring: An arterial intubation was performed into the femoral artery and was monitored with a blood pressure sensor (PT-102) to record the blood pressure and the heart rate by the BL-420F system.

Respiration monitoring: A bandage type respiratory sensor (HX200) was fixed to the chest of the dogs, and the frequency and amplitude of their respiration were recorded with the BL-420F system.

Exposure of suboccipital muscles: The superficial structures were reflected layer by layer until the suboccipital muscles were exposed.

#### Electrical stimulation of the superficial lay of the sub occipital muscles (RCDma, OCI)

The BL-420F biological function laboratory system was used for this aspect of the study. Electrical stimulation was set at an output voltage of 10 V, wavelength of 30 ms and 30 Hz, and the electrical stimulation was performed thrice for 10 s in each side of the long and short heads of the RCDma and the OCI muscles, respectively. The CSF pressure, respiration, and blood pressure were all recorded simultaneously.

##### Passive movements of head

The spinous process of the axis (C2) was fixed with a custom made cervical fixation device to stabilize the neck during passive movements of the head. Lateral flexion, flexion–extension and rotations of the head at the atlanto-occipital and atlanto-axial joints were respectively performed 10 times, with the frequency of motion being once per second. The CSF pressure, respiration,and blood pressures were monitored and recorded.

##### Electrical stimulation of the RCDmi muscle

The stimulating electrode was inserted into the left and right side of the RCDmi respectively, and then electrical stimulations were given with a stimulus intensity of 10 V for 10 s, thrice for each side. The CSF pressure, respiration, and blood pressure were observed and recorded simultaneously.

##### Electrical stimulation in the OCI muscle after severing of its underlying connective fibers

In 6 animals, the left OCI muscle was disconnected from its deep structures and the right side was not treated as a control group. Subsequently the OCI was electrically stimulated on the left and right side respectively, as the CSF pressure, respiration, and blood pressures were monitored and recorded.

##### Euthanizing the animals

The experimental dogs were euthanized by air embolism under anesthesia (3% pentobarbital sodium, 1 ml/kg).

##### Collection and analysis of data

The experimental data was recorded before and after the electrical stimulations by using the Biological function laboratory system machine, and peak value of pressure wave was collected. The data were recorded as: Mean ± SD and analyzed by paired samples t-test and rank sum test (Wilcoxon signed-rank), P < 0.05 was chosen to be a statistically significant result.

### Ethics approval and consent to participate

All animal experiments conformed to ethical requirements of the school.

### Limitations

The experiment was performed under anesthesia, which may be different from the physiological condition; Because of the great difficulty of surgery, the number of cases of myodural bridge disconnection was relatively small, so statistical analysis cannot be realized.

### Approval for animal experiments

All of the described experiments were approved by the Animal Experimental Center of Dalian Medical University. The present study was designed, performed, and reported according to the principles of the ARRIVE guidelines.

### Statement

All methods were carried out in accordance with relevant guidelines and regulations. All experimental protocols were approved by Dalian medical university.

## Results

### The results of anatomical dissection of the suboccipital region

**Figure 3:**
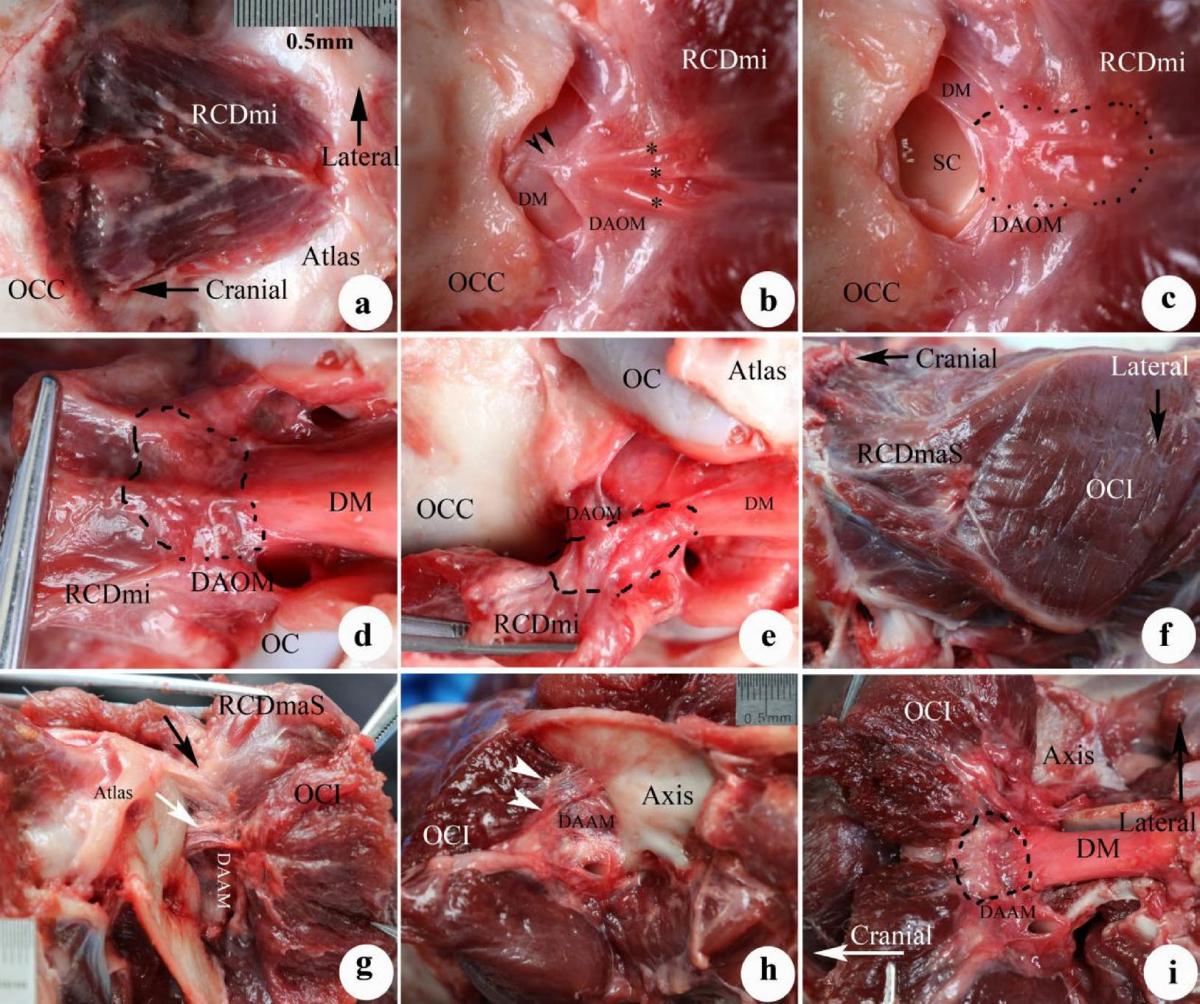
The MDB at the dorsal atlanto-occipital or atlanto-axial interspaces. The RCDmi overlay the dorsal atlanto-occipital interspace (Fig. 3a). When the RCDmi was reflected and stretched caudally the DAOM was severed along the posterior margin of occipital bone, and a fibrous connection was observed between the RCDmi and the DAOM (trigeminy stars), and also between the DAOM and the SDM (double arrowheads)(Fig. 3b). After the DM was cut along the margin of foramen magnum, and the RCDmi was reflected caudally, it was found that the DM was stretched dorsally via the MDB (enclosed by the dotted line) (Fig. 3c). On the other hand, the connective tissues (enclosed by the dotted line) among the RCDmi, the DAOM, and the DM were observed from the caudal side when the posterior arch of the atlas and the vertebral lamina were removed and the RCDmi was reflected to the cephalic side (Fig. 3d). At the lateral side of the dorsal atlanto-occipital interspace, the posterior arch of the atlas and the vertebral lamina of the axis were removed and the RCDmi was reflected laterally, and the connective tissues (enclosed by the dotted line) among the RCDmi, the DAOM, and the DM were again observed (Fig. 3e). Between the atlas and axis, the short head of the RCDma (RCDmaS)and the OCI passed over the dorsal atlanto-axial interspace (Fig. 3f). The fibrous connections between the OCI and the DAAM (white arrow), and between the short head of the RCDma and the DAAM (black arrow) were exposed in cranial aspect (Fig. 3g) and in the caudal aspect (double arrowheads) (Fig. 3h). After the lamina of the axis was removed and the OCI was reflected cranially, the dense connective tissue (enclosed by the dotted line) among the OCI, DAAM, and DM was observed (Fig. 3i). OCC: Occipital bone, RCDmi: Rectus capitis dorsal minor, DAOM: Dorsal atlanto-occipital membrane, DM: Dura mater, SC: Spinal cord, MDB: Myodural bridge, OC: Occipital condyle. OCI: Obliquus capitis inferior, RCPma S: Short head of the rectus capitis dorsal major, DAAM: Dorsal atlanto-axial membrane.

In the suboccipital region, three suboccipital muscles were identified, the RCDma and RCDmi, and OCI. The RCDma was observed to have long and short heads (Fig. 3a,f).

#### MDB in the dorsal atlanto-occipital interspace

The RCDmi was observed to cross over the dorsal atlanto-occipital interspace (Fig. 3a), giving off dense connective tissues ventrally that inserted into the DAOM. The DAOM was observed to be tightly fused to the SDM via dense connective tissues fibers (Fig. 3b-e).

#### MDB in the dorsal atlanto-axis interspace

The short head of the RCDma as well as the OCI were both observed to pass over the dorsal atlanto-axial interspace. The short head of the RCDma was found to cover the small central part of this space and the OCI covered the large lateral part of this space (Fig. 3f). Deep to these two muscles, dense connections were found between the short head of the RCDma and the central part of the DAAM and between the OCI and the lateral part of the DAAM (Fig. 3g,h). Subsequently, when the dorsal arch of atlas was removed to expose the SDM, dense connective tissue bridge was observed extending from the OCI to the SDM via the DAAM (Fig. 3i).

### The histological results of connections between the dorsal occipital muscles and the SDM

#### Histological staining results

**Figure 4:**
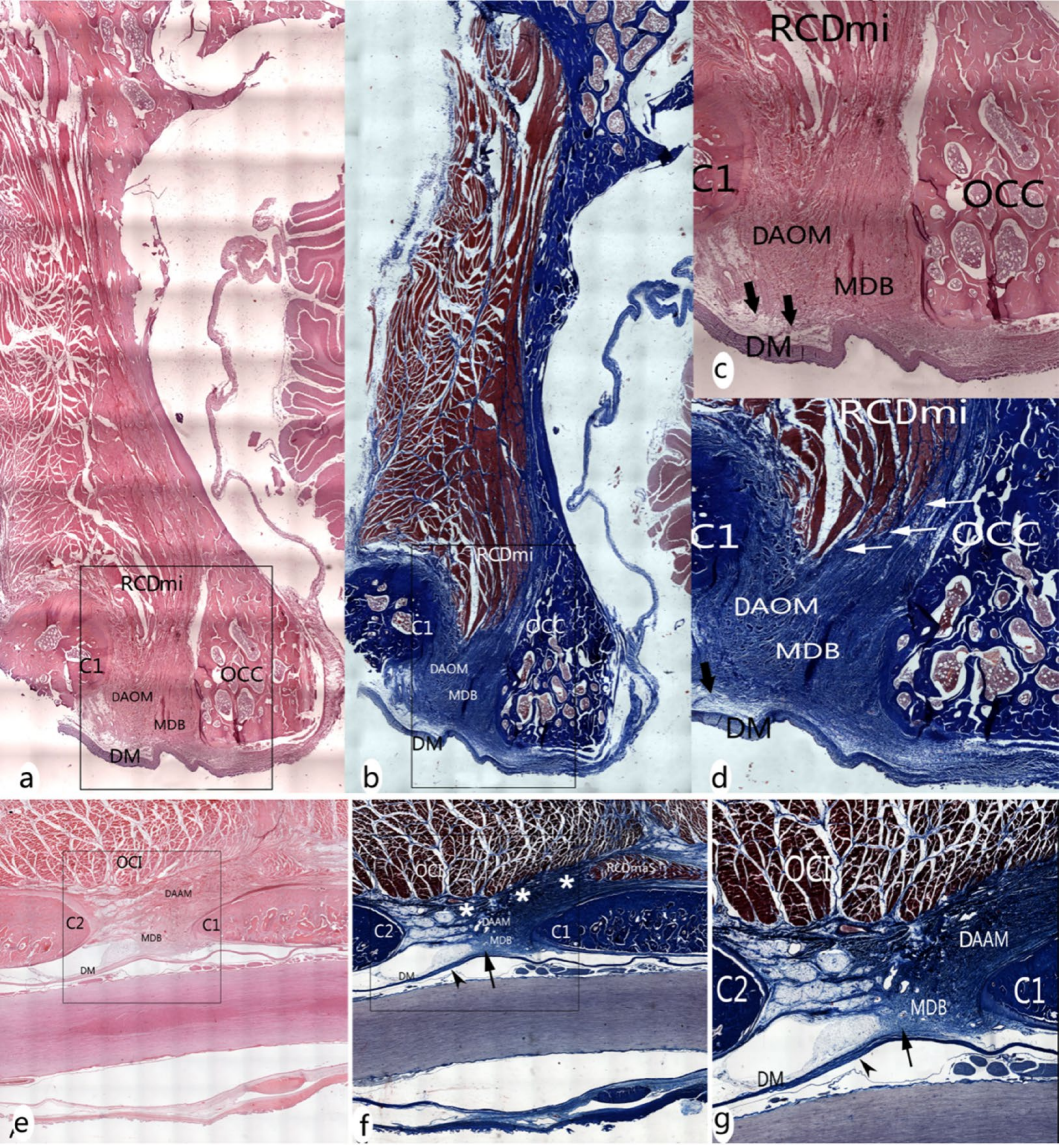
The MDB showed in sagittal section of the dorsal atlanto-occipital or atlanto-axial regions, with H&E stained (**a**, **c**, ** e**) and Masson stained (figure** b**, **d**, **f**, **g**). Abundant dense connective tissues (white arrows in Fig. 4d) were found to be continued with the muscular bundles of the RCDmi and inserted into the DAOM. And then dense fibrous tracks (thick arrows in Fig. 4c,d) from the ventral side of the DAOM connected with the SDM. Meantime, abundant dense connective tissues (white stars in Fig. 4f) were found to be originated from the caudal end of the short head of RCDma and the ventral side of the OCI and inserted into the DAOM. And then the ventral fibers of the dense part of the DAAM are connected to the SDM (black arrow in Fig. 4f,g), where the SDM appeared multilayer structure and became thick caudally (arrowhead in Fig. 4f,g). OCC: the occiput, C1: the atlas, C2: the axis; DAOM: Dorsal atlanto-occipital membrane; DAAM: Dorsal atlanto-axial membrane; RCDmi: Rectus capitis dorsal minor; OCI: Obliquus capitis inferior; RCPma S: Short head of the rectus capitis dorsal major; MDB: the myodural bridge; DM: the spinal dura mater.

At the level of the dorsal atlanto-occipital interspace, it was found that abundant dense collagen fibers from the ventral side of the RCDmi were inserted into the DAOM. The DAOM subsequently gave off collagen fibrous clusters from its ventral side and connected to the SDM (Fig. 4a-d).

At the dorsal atlanto-axial interspace, the cranial part of the DAAM was composed of abundant concentrated dense connective fibers, whereas the caudal part of the DAAM evidenced loose fibers, having scattered parallel running collagen fibrous bundles (Fig. 4e-g).

Dorsal to the DAAM, numerous fibrous collagen bundles were found originating from the ventral aspect of the OCI and dorsal end of the short head of the RCDma and inserted into the cranial dense part of the DAAM, and continuously the DAAM gave off abundant collagen fibers and then fused with the SDM. (Fig. 4e-g).

#### The results of observation under the SEM

**Figure 5:**
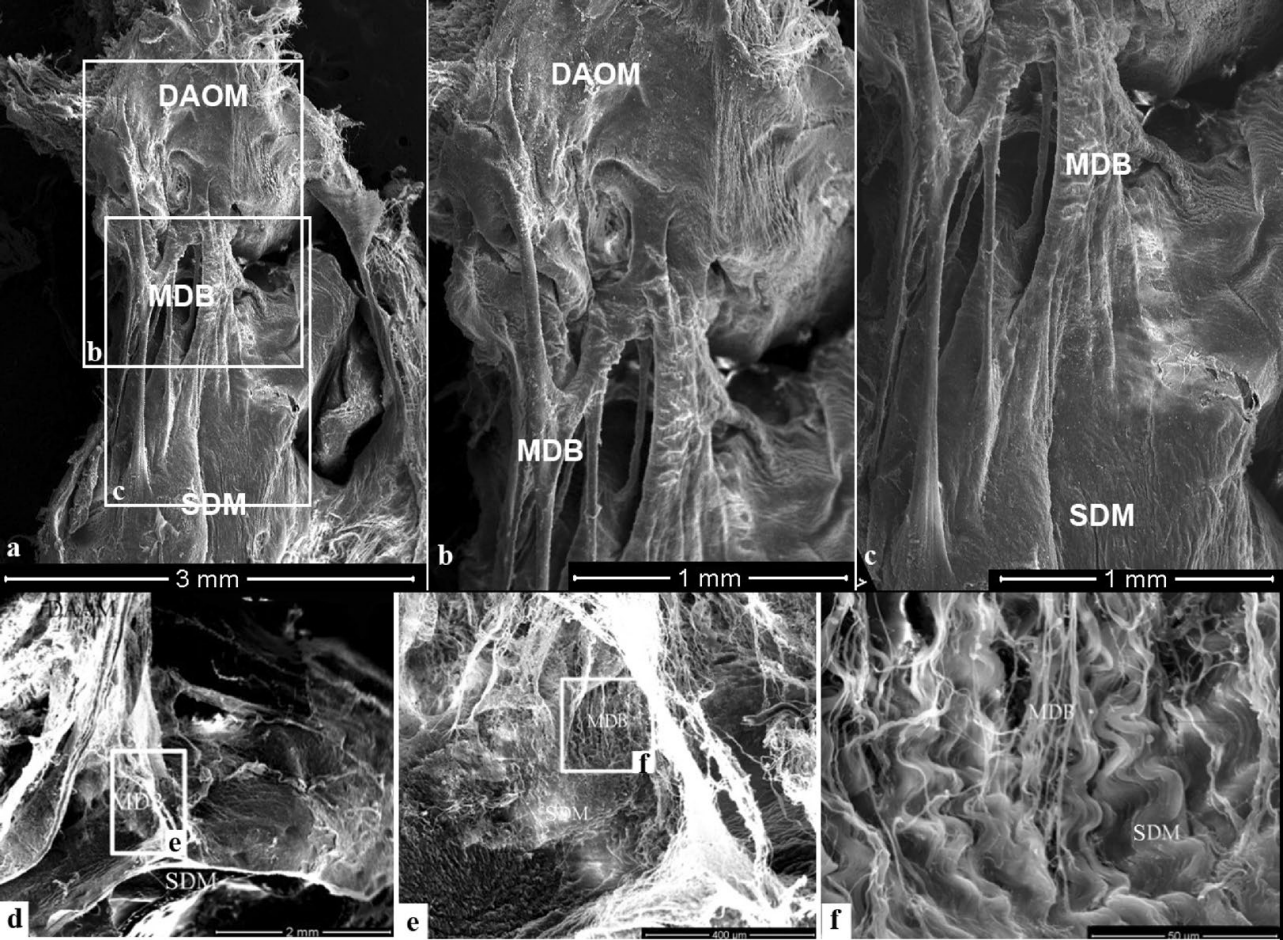
Connections between the DAOM and SDM or between the DAAM and SDM showed under the SEM. Figure (**b**) and (**c**): Magnification of the boxes in figure (**a**). Figure (**e**): Magnification of the box (**e**) in figure (**d**). Figure (**f**): Magnification of the box (**e**) in figure (**e**). Abundant cord-like tissues were a terminal part of the MDB, connecting the DAOM to the SDM (Fig. 5a). These connective cords were fused compactly with the DAOM (Fig. 5b) and the SDM (Fig. 5c) respectively. The DAAM gave off bundle of plexiform collagen fibers (Fig. 5d), which were woven into the collagen fibers array of SDM (Fig. 5e,f), thus anchoring the SDM to the DAAM. DAOM: Dorsal atlanto-occipital membrane; DAAM: Dorsal atlanto-axial membrane; MDB: Myodural bridge; SDM: Spinal dura mater.

For convenience of observation, the DAOM and SDM were separated a little apart and then lots of cord-like structures were found connecting them. These plexiform cords were originated from the ventral side of the DAOM and then fused together with the SDM (Fig. 5a-c). Between the DAAM and the SDM, masses of fibrous clusters were observed. They were given off by the DAAM and then interwoven into the collagen fibrous array of the SDM (Fig. 5d-f).

### Results of the tensile experiment

The RCDmi’s MDB was inserted into the SDM covering about 47.58 ± 10.80 mm^2^. The fracture force of the MDB was 7.55 ± 1.74 N, and the ultimate tensile strength amounts to 0.16 ± 0.04 MPa. The RCDma and OCI’s MDB was insert into the SDM covering 18.60 ± 11.62 mm^2^. The fracture force of the MDB was 13.80 ± 10.43 N, and the ultimate tensile strength 0.82 ± 0.57 MPa.

### Results of CSF pressure monitoring

#### CSF pressure changes during the electrical stimulation of the selected suboccipital muscles

**Figure 6:**
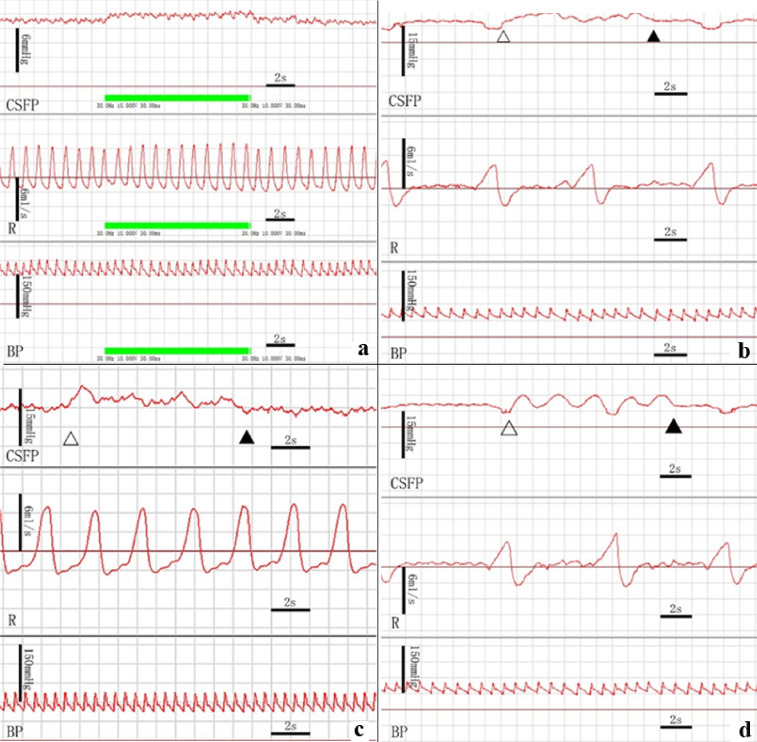
Measurements of CSF pressure during electrical stimulation of the suboccipital muscles or during passive head movements. The CSF pressure was significantly increased when the OCI muscle was stimulated but no change occurred in the blood pressure and respiration rate (Fig. 6a). And a rise and waveform change of CSFP appeared during the lateral head flexion (Fig. 6b), head rotation (Fig. 6c), and head flexion and extension (Fig. 6d), without changes in the respiration and blood pressure. CSFP: Cerebrospinal fluid pressure, R: Respiration, BP: Blood pressure, Hollow Triangle: the beginning of passive motion of the head, Filled Triangle: the end of passive motion of the head.

The results showed that the CSF pressure of the specimens were significantly increased from 12.41 ± 4.58 mmHg to 13.45 ± 5.16 mmHg during electrical stimulation of the OCI (Fig. 6a, Table 1). But when the OCI was detached from the DAAM as a negative control group, CSF pressure of this group was not changed obviously during the electrical stimulation (Table 2). Furthermore, the long and short heads of RCDma, and the RCDmi were stimulated electrically. There were no significant changes in CSF pressure during their electrical stimulation.

**Table 1:** The effects of electrical stimulation of muscles on the CSF pressure (CSFP).

	N	CSF pressure (mmHg)		T value	P value (both sides α = 0.05)
		Before stimulations	During stimulations		
OCI	26	12.41 ± 4.58	13.45 ± 5.16	3.589	0.001
Long head of RCDma	25	12.59 ± 4.61	12.93 ± 4.50	2.042	0.052
Short head of RCDma	9	11.18 ± 5.36	11.68 ± 5.81	1.981	0.083
RCDmi	8	14.33 ± 5.06	14.56 ± 5.56	0.636	0.545

**Table 2:** The role of the myodural bridge in OCI affecting the CSF pressure (n = 6).

	CSF pressure (mmHg)		Increase value (mmHg)	P value
	Before stimulations	During stimulations		
Before detached from the DAAM	12.14 ± 4.11	14.92 ± 5.18	2.10 ± 1.58	0.028
After detached from the DAAM	12.37 ± 3.41	12.34 ± 3.51	− 0.03 ± 0.58	0.917
P value	–	–	0.028	–

#### CSF pressure changes during passive movements of head

It was found that the CSF pressures were increased from 11.08 ± 4.21 to 12.14 ± 4.11 mmHg during the lateral flexion, from 10.73 ± 3.83 mmHg to 11.83 ± 3.88 mmHg during the head rotation, and from 11.25 ± 3.94 mmHg to 12.48 ± 4.08 mmHg during the head flexion–extension respectively. A significantly increasing pressure of CSF appeared during each of the movements (Fig. 6b-d, Table 3).

**Table 3:** The effect of passive head movement on CSF pressure (CSFP).

	N	CSFP(mmHg)		T value	*P* value
		Before motion	During motion		
Head rotation	12	10.73 ± 3.83	11.83 ± 3.88	6.577	0.000
Flexion–Extension	14	11.23 ± 3.94	12.48 ± 4.08	3.912	0.002
Lateral Flexion	14	11.08 ± 4.21	12.14 ± 4.11	4.578	0.001

## Discussion

In the recent 30 years, the MDBs have been discovered constantly between the suboccipital muscles and the upper cervical spinal dura mater (SDM) in the occipitocervical junction in human. The MDB of RCPmi is located in the posterior atlanto-occipital interspace^1,11,13,14,21–25^. And the MDBs of the RCPma and OCI are connected with the spinal dura mater through the posterior atlanto-axial interspace^2,3,13,22^. Further more the nuchal ligament (NL) was also found to participate the MDB at the posterior atlanto-axial and atlanto-occipital interspaces respectively^12,26–29^. Up to now, the MDB has been proved to be a constant anatomical structure in the occipito-cervical junction. But its function is still unclear. Recently, a new functional hypothesis was proposed that the MDB complex might be one of the sources of power for CSF circulation^12–15,22^.

In this study, combining the multi-methods of gross dissection, histology, and scanning electoral microscope, it was provided that the MDB was compose of abundant dense connective fibers existing between the dorsal occipital muscles (the RCDmi, the short head of RCDma, and OCI ) and the SDM. Recently, Zheng et al.^30^ suggested that the MDB might be a common existence and a normal anatomical structure in mammals. The present animal study provides further evidence for the ubiquity of MDB in mammals.

Based on the MDB structure, the tensile strength of the MDB was managed to be measured. The rupture of the myodural bridge complex (MDBc) occurred at site between the DAOM (or DAAM) and the SDM, which may be the weakest part of the MDBc. The ultimate tensile strength (UTS) of the MDB at the dorsal atlanto-occipital and atlanto-axial interspaces are 0.16 ± 0.04 MPa and 0.82 ± 0.57 MPa respectively, and the latter is obviously stronger than the former. It has been reported that the UTS of the arterial wall is 0.5–1.72 MPa^31^. Although the MDBs are different from the arterioles in structure, the UTS of the MDB is similar to that of the arteriole. This allows us easily to perceive the strength of the dural bridge by comparing the MDB with the arterioles in the epidural space.

The dural is a membranous structure, with cerebrospinal fluid in the dural sac, venous plexus and loose connective tissue in the epidural space. The resistance of the dura movement would be small, and the UTS of the MDB attached to the dura mater may be enough to complete the bridge function of pulling the dura mater. And the strength of the dura mater is within the range of 3.28–7.86 MPa^32^, with one order of magnitude stronger than that of the MDB, and this may accord with the primary and secondary relationship between structures and is also necessary to ensure the structural safety of the dura mater. In addition, tendons and ligaments are tough soft tissue structures^33^, and the UTS of the MDB attached to the dura mater is 40 to 2000 times weaker than tendons or ligaments. However, their physiological functions are also significantly different.

This study defines the mechanical properties of the MDB and provides data and method for further investigation on the biomechanical characteristics of the MDB.

In this study, we demonstrated the effect of stimulated contractions of the suboccipital muscles and passive movements of the atlanto-occiptal and atlanto-axial joints on the CSF pressure. The results showed that the electrical stimulation of the OCI caused a significant increase in the CSF pressure, while this effect was eliminated by cutting the myodural bridge of OCI in advance. Also in vivo remaining dorsal occipital muscles intact, there was an obvious rise in the CSF pressure during passive head rotations. Head rotation mainly occurs at the atlanto-axial joint, where the OCI muscle is involved. Combining anatomical and physiological results, it was proved that, during head rotation, the contraction of the OCI is probably transferred to the SDM via the MDB, and repeated traction on the spinal dural during head rotations might lead to reciprocating changes of geometrical shape of the upper cervical spinal dura capsule and then result in the subarachnoid space pressure increase with some kind of dynamic mechanism.

Moreover, the present study found that passive head movements of lateral flexion and flexion–extension all made significant increase of the CSF pressure. During these kinds of head motion, one or more dorsal occipital muscles were involved at the occipito-atlantal joint, and the short head of RCDma and the RCDmi might pull the spinal dura capsule via their MDBs, and then cause a rise in the CSF pressure. Nevertheless, there was no obvious change in the CSF pressure when active electrical stimulation of the short head of RCDma or the RCDmi was performed. The RCDmi and the short head of RCDma are small muscles, and they could not move the the occipito-atlantal joint alone. So during their active electrical stimulation, they produced only isometric contractions and thus a negligible pull on the SDM was present without effect on the CSF pressure.

Generally, the present results provide direct evidence that the MDB complex is one of the power sources for CSF circulation. Additional in clinic, it also sheds light on the possible relationship between pathologic suboccipital muscles and chronic headache. Clinical studies have shown that the dysfunction of the suboccipital muscles under various kinds of pathologic status might be correlated with chronic cervical headache^14,17–20^. The role of the suboccipital muscles via the MDB in CSF circulation might be its possible mechanism.

## Conclusion

The myodural bridge exists in the suboccipital region of dogs and has a tensile strength similar to the walls of small artery. The head movements are an important factor affecting CSF pressure, and the MDB of the suboccipital muscles may play a key role this process. The present study provides direct evidence to support the hypothesis that the MDB may be an important power source for CSF circulation.

## Data Availability

All data generated or analyzed during this study are included in this article. All the data was obtained and documented via the described experiments.
